# Minimally Invasive Surgery for Colorectal Cancer: Past, Present, and Future

**DOI:** 10.1155/2011/490917

**Published:** 2011-08-18

**Authors:** J. Holder-Murray, E. J. Dozois

**Affiliations:** Department of Colon and Rectal Surgery, Mayo Clinic, Gonda 9-205, 200 First Street SW, Rochester, MN 55905, USA

## Abstract

A rapid progression from conventional open surgery to minimally invasive approaches in the surgical management of colorectal cancer has occurred over the last 2 decades. Initial concerns that this new approach was oncologically inferior to open surgery were ultimately refuted when several prospective randomized trials concluded that laparoscopic colectomy could achieve similar oncologic outcomes to open surgery. On the contrary, level 1 data has not yet matured regarding the oncologic safety of minimally invasive approaches for rectal cancer. We review the published literature pertaining to the evolution of minimally invasive techniques used to treat colorectal cancer surgery, including barriers to adoption, and the prospects for future advances related to innovative techniques.

## 1. Introduction

Since the introduction of laparoscopic surgery, minimally invasive techniques have been broadly applied across multiple specialties for both benign and malignant conditions. The adoption of laparoscopic cholecystectomy by general surgeons following its inception in 1987 marked an important transition away from more invasive open techniques [1]. In 1991, Jacobs et al. reported the first laparoscopic colectomy, and the enthusiasm for laparoscopic colectomy grew when recovery benefits for patients became more apparent [2]. Though many surgeons soon became comfortable with laparoscopic colectomy for benign disease, the application of minimally invasive surgery (MIS) to malignant colorectal disease was slow due to oncologic concerns [3]. With time, numerous randomized controlled trials comparing laparoscopic to open surgery for colon cancer were published, clearly demonstrating that in experienced hands, appropriate oncologic resections can be performed and produce results equivalent to the open techniques [4–7]. In contrast, the outcomes of MIS for rectal cancer have not yet been definitively studied, but large randomized, multicenter trials are underway. Because rectal cancer surgery demands more technical expertise (total mesorectal excision, low pelvic anastomosis) than colectomy, many have concerns that oncologic principles may be compromised when rectal cancer is treated laparoscopically, leading to worse oncologic outcomes. 

In addition to oncologic concerns, the widespread application of laparoscopic techniques to colorectal cancer was also limited by the substantial learning curve encountered for many surgeons, especially those not trained in MIS. Hand-assisted techniques introduced in the early 1990s made an attempt to overcome some of these limitations, providing an overlap between open and laparoscopic techniques and thus facilitating the transition from open to MIS for many surgeons [8]. 

The acceptance of minimally invasive procedures by both patients and surgeons has led many surgical innovators and industry to develop new technology with the goal of even less invasive approaches. The advent of the single-incision laparoscopic surgery (SILS) devices has allowed fewer incisions [9]. Robotic techniques popularized in other specialities, such as urology, have been applied to surgery for rectal cancer to overcome the limitations of conventional laparoscopy in the confined working space of the pelvis [10, 11]. The clinical application of natural orifice transluminal endoscopic surgery (NOTES) to colorectal disease has not yet fully transpired, though there have been major advances as instrumentation improves and transitional techniques allow natural orifice specimen extraction following laparoscopic colectomy [12–15]. 

The aim of this paper is to review the published literature regarding the evolution of MIS for colorectal cancer including the barriers to adoption, current status, and prospects for future advances related to innovative techniques.

## 2. MIS for Colon and Rectal Cancer: Where Have We Been?

After the initial description in 1991, several reports of MIS for colorectal cancer were described. Significant concerns regarding this approach surfaced when minimally invasive techniques applied to colorectal malignancy lead to increased surgical complications and worse cancer outcomes compared to conventional open approaches. An early report, using minimally invasive techniques for benign colorectal disease, showed a significantly high rate of serious complications (18%), including inadvertent enterotomies, intraoperative hemorrhage, anastomotic leaks, and pelvic abscesses [16]. When MIS was used to treat colorectal cancer, several papers noted early wound or trocar site recurrences, including one case series documenting a 21% rate [3]. With a less than 1 percent wound implantation rate for open surgery, serious concerns were raised as to the possibility that poor oncologic results were due to a combination of poor technique and abnormal distribution of malignant cells secondary to pneumoperitoneum [17]. Further concerns that laparoscopic techniques may be problematic to cancer patients arose when some studies demonstrated statistically significant worse cancer-specific survival in patients who had conversion from laparoscopic to open surgery [18, 19]. Moloo et al. described decreased survival at 2 years of 76% from 87% for all stages (*P* = 0.02) of colorectal cancer collected from a prospective database of 377 consecutive laparoscopic patients. In the same cohort, at 5 year followup, there was a trend toward decreased overall survival in converted patients (61.9% versus 69.7%, *P* = 0.077). Chan et al. showed an increased local recurrence rate at 3 year followup of 9.8% in the laparoscopically converted group as compared to 2.8% in open patients (*P* = 0.03). 

The oncological concerns raised in early reports provided a compelling argument to study the question of oncologic equivalence between the open and laparoscopic approach to colorectal cancer in a controlled fashion. In the early 1990s, several multicenter prospective randomized controlled trials comparing laparoscopic and open surgery for colorectal cancer were initiated. Ultimately, seven large-scale trials compared laparoscopic and open colectomy for colon carcinoma and examined short-term and long-term outcomes. These trials included the Clinical Outcomes of Surgical Therapies (COST) trial funded by the National Cancer Institute in the United States, the Conventional versus Laparoscopic-Assisted Surgery in Colorectal Cancer (CLASICC) trial in the United Kingdom, the Colon Cancer Laparoscopic or Open Resection (COLOR), a multicenter European trial, the Barcelona trial, and several others [20–26]. The main focus of these trials was oncologic outcomes, but short-term outcomes, quality of life, and safety were also evaluated. The CLASICC trial was the only large trial that also evaluated MIS in rectal cancer.

## 3. MIS for Colon Cancer: Where Are We Now?

Though modest in early studies, the short-term patient-related advantages of laparoscopic surgery have now been confirmed and are significant over the open approach. The Minimally Invasive Colorectal Resection Outcomes (MICRO) review identified 22 randomized controlled trials and 66 cohort series for benign and malignant colorectal disease [27]. Laparoscopic colectomy results in significantly lower pain scores and analgesia requirements, estimated blood loss, return of bowel function, and length of stay. Numerous other trials, including the COST, COLOR, and CLASICC trials, examining short-term outcomes following laparoscopic colectomy for colorectal cancer have confirmed these findings [20–26, 28]. Several studies have also identified a decreased rate of postoperative morbidity including fewer wound infections [21, 23, 27, 29]; this was recently reinforced by a large trial from the National Surgical Quality Improvement Program (NSQIP) database of over 10,000 patients identifying decreased incidence of wound infection following laparoscopic colectomy (9.5% versus 16.1%, *P* < 0.001) [30]. Quality of life has been assessed in several trials and results varied from no difference to favoring improved quality of life in laparoscopic colectomy [31]. 

The initially cited oncologic concerns of laparoscopic colectomy for colorectal cancer were later dispelled when surgeons trained in appropriate laparoscopic oncologic resection performed operations in the trial setting. Major trials, including the COST, CLASICC, and COLOR trials, examined tumor specimens and reported long-term data on recurrence and survival. The surgical specimens were evaluated, and parameters such as lymph node yield, circumferential radial margins, and longitudinal margins were quantified. No trial identified statistically significant differences in lymph node yield [20–26] or resection margins [20, 22, 26]. This initial evidence allayed some concerns regarding oncologic resections, but the long-term measures for recurrence and survival were still unknown. Trial data matured, and more evidence accumulated confirming similar recurrence patterns and rates between laparoscopic and open colectomy. Local recurrence, distant recurrence, and wound or port site metastases were the same between groups [4, 5, 7, 24, 32–34]. Disease-free and overall survival in long-term followup (up to 7 years) is equivalent [4, 5, 7, 32–34]. The concern that conversion from laparoscopic to open surgery in patients with colon cancer may lead to worse oncologic outcomes was not seen when 5-year COST trial data showed no statistical difference in these two groups [5] (see Table 1).

Despite evidence demonstrating improved short-term outcomes of laparoscopic colectomy and oncologic equivalence, widespread implementation of this technique was slow. The lack of formalized training, outside single-day laparoscopic training courses, and the significant learning curve for straight laparoscopic techniques likely represented significant barriers to adoption. As hand-assisted laparoscopic surgery grew in popularity, a more widespread adaptation with fewer conversions to open surgery occurred in part due to a shorter learning curve with this technique. Three randomized controlled trials have been performed to compare a hand-assisted technique to a laparoscopic technique including patients with both benign and malignant disease, all demonstrating decreased rates of conversion to open surgery [35–37]. A recent meta-analysis compiling 13 studies demonstrated decreased operative times and decreased open conversion rates with an hand-assisted approach [38]. There were no differences in short-term clinical outcomes or oncologic resection results. A recent study by the Mayo Clinic prospectively analyzed the use of hand-assisted surgery in a minimally invasive colorectal practice and found that when applied to a center performing large volumes of laparoscopic surgery, hand-assisted techniques were responsible for more complex procedures to be done laparoscopically [39]. This technique is a minimally invasive approach that has been helpful for surgeons to transition from open to laparoscopic colectomy, especially if they have had little previous laparoscopic experience. Moreover, this technique has allowed a MIS approach in patients otherwise not previously considered candidates (obese, adhesions). 

As surgeon experience increased and as more studies demonstrated that laparoscopic colectomy for benign and malignant disease is an acceptable alternative to open surgery, the overall ratio of laparoscopic to open colectomies in the United States has increased. A recent analysis from 2000 through 2004 demonstrated an increasing incidence of laparoscopic colectomy from 3% to 6.5% nationally with increased rates of laparoscopic approaches in urban centers and teaching hospitals [40]. A separate study and database of patients from 2004 through 2006 identified over 32,000 patients, of which 34% underwent laparoscopic colectomy [41]. This trend toward increased laparoscopy has also been influenced by public knowledge and patient demand for this approach, as well as improved and formalized laparoscopic training in residency programs. 

## 4. MIS for Rectal Cancer: Where Are We Now?

The benefits and oncologic results of laparoscopic techniques in rectal cancer surgery have not yet been definitively established. Several trials show similar outcomes as laparoscopic colectomy for colon cancer; however, level 1 data is lacking. Several multicenter randomized prospective trials are ongoing, and data will mature in the near future: American College of Surgeons Oncology Group (ACOSOG) Z6051 in the United States, the COLOR II trial of Europe, Canada, and Korea, and the Japan Clinical Oncology Group (JCOG) 0404 trial [42].

Despite limited randomized multicenter trials, several single institution comparative studies have shown promising results using MIS for rectal cancer. Leung et al. compared laparoscopic to open resection of rectosigmoid carcinoma in over 400 patients [43]. Laparoscopic resection demonstrated improved short-term outcomes, longer operative times, decreased estimated blood loss, equivalent morbidity and mortality, and equivalent long-term results of overall survival, disease-free survival, and local and distant recurrence. In a subgroup analysis of upper rectal cancer lesions undergoing anterior resection, these findings were reiterated [44]. For abdominoperineal resection, Ng examined 99 patients and again demonstrated improved short-term outcomes, longer operative times, equivalent morbidity and mortality, and equivalent long-term results of overall survival, disease-free survival, and local recurrence [45]. Each study demonstrated similar oncologic resection results including equivalent lymph node yield and resection margins. Rullier evaluated his single center data for laparoscopic rectal cancer specifically examining long-term outcomes and resection margins for completeness of a total mesorectal excision (TME). In the largest series of 471 patients, 92% of laparoscopic excisions versus 94.8% of open excisions had an R0 resection (*P* = 0.22), with no difference of local recurrence (3.9% versus 5.5%, *P* = 0.37) and disease-free survival (82% versus 79%, *P* = 0.52) at 5 years [46]. Upon examining mid to low rectal cancer specimens of T1/T2 or T3 tumors (17% versus 83%) following laparoscopic excision, 88% had an intact TME with R0 resection in 93% of cases [47]. Only one randomized trial from Brazil of 28 patients demonstrated decreased lymph node yield [48]. A meta-analysis of short-term outcomes confirmed the findings of faster return of bowel function, decreased analgesia use, and decreased length of stay in laparoscopic patients [49]. Operative times were longer with similar lymph node yield and rates of positive circumferential radial margins. 

The only multicenter randomized trial to date with oncologic outcome data examining laparoscopic rectal cancer is the CLASICC trial, which included both colon and rectal adenocarcinoma patients [6, 22, 32]. A subgroup analysis of the laparoscopic rectal dissection patients demonstrated a concerning trend toward increased positive circumferential radial margins (12% versus 6%, *P* = 0.19) following anterior resection with equivalent distal margins and lymph node yield for all rectal cancers [6]. There was no difference in the overall survival, disease-free survival, or local recurrence at 3 years (see Table 2).

Pelvic dissection for rectal carcinoma is technically challenging due to limited visual exposure, operating within a narrow confined space, and the possibility of local invasion to surrounding structures. Laparoscopic dissection may overcome some of these difficulties by offering improved visual angles and magnification of the pelvis. Unfortunately, several technical challenges must be overcome for the success of laparoscopic rectal dissection. Adequate rectosigmoid retraction must be achieved by the operative assistant in order to provide exposure and tissue tension for dissection. Further, the narrow confines of the pelvis limit the mobility of standard laparoscopic instruments. If an anterior resection is performed, the current laparoscopic stapling devices are difficult to maneuver into a narrow pelvis and position for a perpendicular staple line, leading to suboptimal distal rectal transection from multiple staple firings. 

Robotic surgical platforms have been proposed as a way to overcome the limitations described above for laparoscopic rectal cancer surgery. Although there are no randomized controlled trials for robotic TME for rectal cancer, several studies demonstrate similar operative duration, intraoperative and postoperative complication rates, and short-term outcomes when compared to laparoscopic controls [10, 11, 50–54]. Robotic TME has similar distal and circumferential radial margins when compared to laparoscopic controls, therefore demonstrating feasibility for this technique [10, 50–54]. Some reports have demonstrated similar overall survival, disease-free survival, and local recurrence at 3 years when compared to laparoscopic approaches [6, 10, 50]. Despite similar operative times, there is a trend toward increased cost with robotic approaches [51]. More rigorous studies are still needed to determine the utility and efficacy of robotic surgery for rectal cancer.

## 5. MIS for Colorectal Cancer: Where Are We Going?

The transition from invasive open surgery to laparoscopy has triggered a surge of innovative techniques and technologic advances for application to many specific surgical procedures. These new techniques and technologic advances attempt to overcome some of the limitations of standard laparoscopy, decrease patient morbidity, and improve cosmesis. For example, single incision laparoscopic surgery (SILS) limits the number of port sites needed and therefore decreases tissue trauma and improves cosmesis. Natural orifice transluminal endoscopic surgery (NOTES) offers an alternative specimen extraction site in order to eliminate abdominal wall incisions necessary for specimen extraction. Innovative devices such as new port designs, instrumentation with increased mobility, and robotics allow the transition toward newer less invasive surgical techniques. Clinical trials evaluating each technique are necessary to ensure continued patient safety and oncologically appropriate surgery. 

SILS (also referred to as SPA (single-port access) or SPLS (single-port laparoscopic surgery)) is a new addition to the colorectal surgeon's armamentarium. The first description of the application of SILS to general surgery was a cholecystectomy in 1997 [55]. Since its inception, SILS has also been applied to appendectomy, splenectomy, herniorrhaphy, tubal ligation, and more. To date, there have been only a handful of case reports of SILS application to colorectal surgery; however, initial descriptions appear promising. In 2008, 2 groups reported their initial experience with SILS right colectomy [56, 57]. Since these reports, two small series have now published outcomes for SILS hemicolectomy in benign and malignant disease [58, 59]. Case-matched analysis comparing SILS and laparoscopic colectomy have revealed similar operative time, length of stay, lymph node yield, and complication rate [58]. 

As new limited access techniques evolve, we must be cautious that limited access to the abdomen does not result in risky or inadequate surgery. Patient selection and specific instrumentation are also essential to successful SILS colectomy. Given the learning curve required to operate under mobility constraints, ideal candidates for SILS colectomy have few or no prior surgeries and a BMI <30. Essential instrumentation includes an elongated laparoscope such as a bariatric length laparoscope or a flexible tip laparoscope in order to move the camera away from the operating hands and, therefore, prevent crowding and allow working instrument triangulation. SILS often requires the surgeon's hands to cross each other during dissection creating internal and external conflicts; this requires adaptation and skill to adopt. Robotics may overcome this technical limitation of single-port surgery: the arms of the robot can be positioned at varying angles and depths, and the robot allows the surgeon to choose the instrument to be controlled by each hand, regardless of the robotic arm that holds the instrument. Robotics in SILS has been successfully performed in an animal model [60].

A transition to NOTES surgery has been increasingly cited in the past year with a partial colectomy performed laparoscopically and the specimen extracted via a natural orifice. This is a technique that limits abdominal trauma to small trocar port sites only. Several recent articles have described specimen extraction transvaginally or transanally with success [12–15]. Franklin et al. described transanal extraction following left hemicolectomy for stage III colorectal carcinoma with good oncologic results [61]. Interestingly, as we reflect on the history of laparoscopic colorectal surgery to the present, it is interesting to note that Jacobs et al. actually utilized transrectal extraction for some of the first laparoscopic colectomy surgeries performed in 1991 [2]. Barriers to transanal or transrectal extraction include anal stenosis, small caliber rectum, and bulky mesentery or specimen. Concerns related to transanal extraction include intraabdominal contamination leading to pelvic sepsis and poor oncologic outcomes. A recent series of 21 patients had no episodes of pelvic sepsis [15]. Transvaginal specimen extraction has proven safe in small series without adverse events [12, 14]; however, this technique excludes women of childbearing age for risk of infertility and women with severe pelvic adhesions. Further evaluation needs to occur regarding long-term dyspareunia from this approach. No randomized data exists studying the long-term oncologic outcomes, including site of tumor recurrence, for natural orifice specimen extraction for colorectal malignancy; however, extracting the specimen via a specimen retrieval bag system to prevent contamination of the tract (similar to a wound protector) adheres to sound oncologic principles and practices already in place for laparoscopic techniques. This laparoscopic/endoscopic surgical platform may require intracorporeal anastomosis, which requires surgeons adept with this technique.

Entirely NOTES colectomy in a live human has yet to be performed. Lacy et al. performed a minilaparoscopic-assisted NOTES sigmoidectomy via a transvaginal technique for sigmoid adenocarcinoma in 2008 [62]. Whiteford utilized cadaveric models to demonstrate feasibility of a radical sigmoid colectomy using a NOTES technique with transanal endoscopic microsurgery (TEM) technology [63]. Although feasible, the mesorectal and retroperitoneal dissection to mobilize the mesorectum from the presacral space may not clearly identify and preserve nerves in this area as the dissection begins inferiorly at the sacrum and works superiorly toward the proximal margin. This will require further investigation if this technique is to be performed in humans. Moreover, the paper describes a blind coupling of the stapler and anvil of an EEA stapler and blind stapler firing. Concerns for this technique include no visualization during the anastomosis and potential twisting or tension on the anastomosis. A handsewn technique utilizing standard TEM principles may obviate these concerns. Two studies in animal models demonstrated NOTES sigmoidectomy via an operating gastroscope [64, 65]. A novel tool for retraction of the sigmoid colon was utilized, that is, a paired magnet technique that incorporates a magnet on the end of a sigmoidoscope and an external magnet. These paired magnets, therefore, allow retraction and movement of the sigmoid colon to facilitate dissection. Further, novel closure devices including endoscopic tacks have been used to close enterotomy defects [66]. 

As these tools and others on the horizon approach clinical use, more devices will be at the surgeon's disposal. The transition to innovative techniques should occur with caution, however. We must prove that there is patient benefit without increasing morbidity. The transition must be safe. Appropriate education should occur for surgeons learning new and dramatically different techniques.

## 6. Conclusions

Minimally invasive surgery for colorectal cancer has been subjected to rigorous scientific evaluation, and due to positive outcomes when done by experienced surgeons, this approach has become the standard worldwide. Level 1 data now supports general feasibility, safety, improved patient-related benefits and oncologic equivalence when compared to open surgery for colon cancer. Though MIS for rectal cancer has been extensively studied in an uncontrolled fashion, multicenter randomized trials are needed to determine oncologic equivalence to open surgery. Innovative approaches to further decrease abdominal wall trauma are currently being tested, and modification to current approaches will likely take place in the near future. The steep learning curve, cost and formalized training continue to be barriers to the widespread application of MIS techniques for colorectal cancer. General and colorectal surgeons must remain fully engaged in the development and application of new technologies and procedures so that surgeons can lead the way into the future while maintaining the patient's interests first.

## Figures and Tables

**Table 1:** Short-term and long-term outcomes of large-scale randomized controlled trials for laparoscopic colectomy compared to open colectomy for colon cancer.

	COST	CLASICC	COLOR	Barcelona	Braga	Milsom	Liang
	[5, 20, 28]	[6, 22, 32]	[4, 26]	[7, 23]	[21, 33]	[25]	[24]
Return of bowel function		=	↓	↓	↓	↓	↓
Pain score						↓	↓
Narcotic use	↓		↓			↓	
Length of stay	↓	↓	↓	↓	↓	=	
OR time	↑	↑	↑	↑	↑	↑	↑
EBL			↓	↓	↓	=	↓
LN yield	=	=	=	=	=	=	=
Circumferential margin +		=	=				
Postoperative morbidity	=	=	=	↓	↓	=	=
Postoperative mortality	=	=	=	=	↓	=	
Quality of life	=	=			↑		
Overall survival	=	=	=	=	=		
Disease-free survival	=	=	=	=	=		
Local recurrence	=	=	=				=
Distant recurrence	=	=	=	=			=
Wound/port recurrence	=	=	=	=	=	=	=

OR: operating room; EBL: estimated blood loss; LN: lymph node. Each outcome recorded is compared to open controls. ↑ or ↓ represents a statistically significant difference related to the outcome; otherwise, = represents no statistical difference.

**Table 2:** Short-term and long-term outcomes of randomized controlled trials for laparoscopic rectal surgery compared to open rectal surgery for rectal cancer.

	Araujo [48]	CLASICC [6, 32]	Ng [45]	Ng [44]	Leung [43]
Return of bowel function			↓	↓	↓
Pain score			=		↓
Narcotic use			↓	↓	↓
Length of stay	=		=	↓	↓
OR time	↓		↑	↑	↑
EBL	=		=	=	↓
LN yield	↓		=	=	=
Circumferential margin +		=	=	=	
Postoperative morbidity	=		=	=	=
Postoperative mortality			=	=	=
Overall survival		=	=	=	=
Disease free survival		=	=	=	=
Local recurrence	=	=	=	=	=
Distant recurrence		=			=

OR: operating room; EBL: estimated blood loss; LN: lymph node. Each outcome recorded is compared to open controls. ↑ or ↓ represents a statistically significant difference related to the outcome; otherwise, = represents no statistical difference.
